# Molecular Dynamics Simulations of DNA-Free and DNA-Bound TAL Effectors

**DOI:** 10.1371/journal.pone.0076045

**Published:** 2013-10-10

**Authors:** Hua Wan, Jian-ping Hu, Kang-shun Li, Xu-hong Tian, Shan Chang

**Affiliations:** 1 College of Informatics, South China Agricultural University, Guangzhou, China; 2 College of Chemistry, Leshan Normal University, Leshan, China; Wake Forest University, United States of America

## Abstract

TAL (transcriptional activator-like) effectors (TALEs) are DNA-binding proteins, containing a modular central domain that recognizes specific DNA sequences. Recently, the crystallographic studies of TALEs revealed the structure of DNA-recognition domain. In this article, molecular dynamics (MD) simulations are employed to study two crystal structures of an 11.5-repeat TALE, in the presence and absence of DNA, respectively. The simulated results indicate that the specific binding of RVDs (repeat-variable diresidues) with DNA leads to the markedly reduced fluctuations of tandem repeats, especially at the two ends. In the DNA-bound TALE system, the base-specific interaction is formed mainly by the residue at position 13 within a TAL repeat. Tandem repeats with weak RVDs are unfavorable for the TALE-DNA binding. These observations are consistent with experimental studies. By using principal component analysis (PCA), the dominant motions are open-close movements between the two ends of the superhelical structure in both DNA-free and DNA-bound TALE systems. The open-close movements are found to be critical for the recognition and binding of TALE-DNA based on the analysis of free energy landscape (FEL). The conformational analysis of DNA indicates that the 5′ end of DNA target sequence has more remarkable structural deformability than the other sites. Meanwhile, the conformational change of DNA is likely associated with the specific interaction of TALE-DNA. We further suggest that the arrangement of N-terminal repeats with strong RVDs may help in the design of efficient TALEs. This study provides some new insights into the understanding of the TALE-DNA recognition mechanism.

## Introduction

TAL (transcriptional activator-like) effectors (TALEs) are secreted by plant pathogenic bacteria that cause diseases in plants [1]–[3]. When TALEs are injected into plant cells, they enter the nucleus, bind to effector-specific sequences and manipulate host gene expression [3]–[5]. The DNA-binding domain of TALEs contains multiple (from 1.5 to 33.5), tandemly repeated units [3]. Each repeat comprises 33∼35 (mostly 34) amino acids and shows high sequence conservation except for the residues at position 12 and 13. The two residues, termed repeat-variable diresidues (RVDs), were found to determine DNA-binding specificity [6], [7]. A simple code was established between RVDs and target DNA bases [6], [7], like Asn/Ile (NI) for recognition of adenine (A), His/Asp (HD) for recognition of cytosine (C), Asn/Gly (NG) for recognition of thymine (T) and so on. On one hand, the TALE-DNA recognition code enables the prediction of DNA target sequences of TALEs [6]–[8]. On the other hand, by using this code TALEs can be customized more easily than other known DNA binding proteins to recognize desired DNA sequences [9], [10]. Engineered TALE proteins have been widely used to genome modifications, such as plants [11], [12] and animals (including humans) [13]–[15]. As a result, the DNA-binding domain of TALEs is considered to be an efficient tool for genetic editing [16], [17].

Because of the advantage from the modular nature of TALE-DNA binding, recently many studies focused on the recognition mechanism of TALE-DNA. In 2010, Murakami et al. reported the first structural data of TALE [18], which was a nuclear magnetic resonance (NMR) structure of 1.5 TAL repeats in the protein PthA. However, the length of 1.5-repeat effector is too short to provide more detailed structural data. In 2012, two groups [19], [20] separately published their structural studies of TALEs. The first group led by Shi et al. determined two crystal structures of an engineered 11.5-repeat TALE dHax3 in both DNA-bound and DNA-free states at 1.8 Å and 2.4 Å resolution, respectively [19]. The second group led by Stoddard crystallized a 3.0 Å structure of the naturally occurring TALE PthXo1 with 23.5 repeats bound to DNA [20]. The two groups both described that the repeats self-associate to form a right-handed superhelix and bind with the DNA major groove. In each repeat, the first residue of RVD (position 12) likely plays a structural role in stabilizing the RVD-containing loop for contacting with DNA, and the specific interaction of TALE-DNA is formed solely by the second residue of RVD (position 13). Recently, another two studies by Shi et al. demonstrated that TALE can also recognize modified bases [21] and bind with DNA-RNA hybrids [22]. The recognition efficiencies of different RVD types were investigated by several studies [23]–[25], strongly indicating that RVDs NN and HD contribute most to overall activities of TALEs. Additionally, some other issues were also frequently discussed, which included a reasonable model for TALE-DNA target search and the possible role of flanking elements in TALE [16], [20], [26], [27]. The N-terminal region was suggested to serve as an active site for DNA binding and subsequent target site recognition [27]. These crystallographic and biochemical studies provided a lot of important structural information about the sequence-specific recognition of DNA by TALEs.

In addition to experiments, theoretical approaches were also applied to explore the TALE-DNA recognition mechanism. Moscou et al. [7] developed a computational method to search DNA sequence for TALE target sites and decided the TALE-DNA recognition code. Cong et al. [28] performed the molecular dynamics (MD) simulations and free-energy perturbation (FEP) calculations to further analyze the guanine specificity of RVD Asn/His (NH). The simulations were based on a fragment of the crystal structure of TALE PthXo1 by Stoddard et al. [20]. By using the Rosetta package, Bradley designed the structural models to predict the interactions for the TALE-DNA system [29]. Additionally, a suite of software tools were provided to the efficient TALE design and target prediction [30], [31]. These works improve our understanding of the TALE-DNA interaction. Nevertheless, the crystal structures of TALE dHax3 by Shi et al. [19], in both DNA-free and DNA-bound states, have not been simulated systemically. The comparison of the two crystal structures revealed that the TALE undergoes a dramatic conformational change upon DNA interaction, which was consistent with the previous small-angle X-ray scattering (SAXS) data [18]. The TALE-bound DNA was found largely in the B-form [19], [20]. Meanwhile, the protein-induced deformability of DNA was often linked to the specific recognition of DNA sequences and transcription activation [32]–[35]. Thus, some detailed questions still need to be solved. What interactions at atomic level are formed between the TALE and the DNA? How do the TALE dynamics affect the recognition and binding of TALE-DNA? Does the DNA have the structural deformability when binding with the TALE? Are there any correlations between the structural change of DNA and the sequence-specific recognition by TALE?

In this paper, in order to answer the above questions, the crystal structures of both DNA-free and DNA-bound TALE dHax3 were analyzed by MD simulations. We investigated the interactions between 11.5 TAL repeats and the DNA, and compared the TALE-DNA interactions from different TAL repeats. In addition, principal component analysis (PCA) and free energy landscape (FEL) methods were applied to probe the functional motions of TALEs and identify the dominant conformational states, respectively. Finally, we calculated the structural deformability of the TALE-bound DNA at the base-pair level, and further suggested the association between the conformational change of DNA and the specific interaction of TALE-DNA.

## Systems and Methods

### The Structures of DNA-free and DNA-bound dHax3 Systems

**Figure 1:**
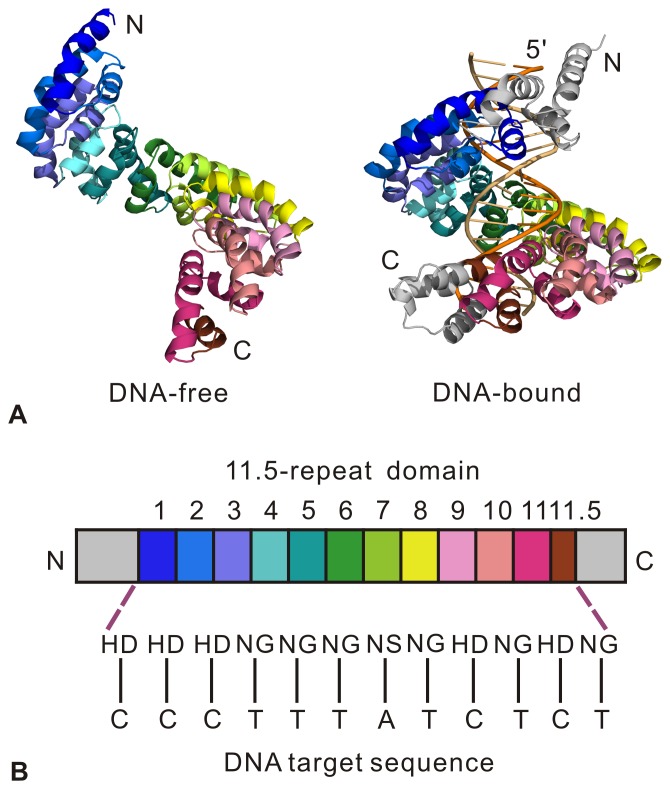
Structures and domain organization (PDB codes: 3V6P and 3V6T). (A) The structures of dHax3 in the DNA-free state (left) and DNA-bound state (right). Each structure contains an 11.5-repeat domain, forming a right-handed superhelical assembly. The 11.5 repeats are colored separately. (B) The 11.5-repeat domain mediates DNA binding. Each repeat recognizes one specific nucleotide by using the RVD residues at positions 12 and 13. Single-letter abbreviations for the amino acid residues are as follows: A, Ala; C, Cys; D, Asp; E, Glu; F, Phe; G, Gly; H, His; I, Ile; K, Lys; L, Leu; M, Met; N, Asn; P, Pro; Q, Gln; R, Arg; S, Ser; T, Thr; V, Val; W, Trp; and Y, Tyr.

The two crystal structures of DNA-free and DNA-bound dHax3 (PDB codes: 3V6P and 3V6T) [19] each contain an 11.5-repeat TALE (see Figure 1 A). The 11.5 TAL repeats form a right-handed superhelical assembly. The DNA-free dHax3 possesses an extended conformation while that of DNA-bound dHax3 is more compact. In the DNA-bound structure, the superhelix wraps itself along the sense strand of DNA duplex and binds in the major groove of DNA. The 11.5-repeat domain confers DNA sequence specificity, with RVD residues of each repeat recognizing one specific nucleotide (see Figure 1 B).

### Molecular Dynamics Simulation

The two simulation systems were set up by VMD 1.9 [36]. Each initial structure was placed in a cubic periodic box filled with TIP3P water molecules. The minimum distance was about 10 Å from the solute unit to the edge of the box. To achieve electroneutrality, 2 Cl^−^ and 29 Na^+^ ions were added to the DNA-free and DNA-bound systems, respectively. Then, the two MD simulations were carried out with the NAMD 2.6 program [37] using the CHARMM27 all-atom additive force field for nucleic acids [38]. The SHAKE algorithm [39] was used to constrain all bonds involving hydrogen atoms, and particle mesh Ewald (PME) [40] method was applied to evaluate electrostatic interactions. Meanwhile, Lennard-Jones interactions were cut off at 12 Å. Each simulation included two stages. (i) The systems were energetically minimized with 20000 steps and then slowly heated over 0.5 ns from 0 K to 310 K. The positions of dHax3 and DNA were restrained with a harmonic constant of 0.1 kcal·mol^−1^·Å^−2^ to keep the stabilization of systems. (ii) The production runs of 20 ns were carried out for each unrestrained system under constant pressure (1 atm) and temperature (310 K) conditions. The pressure and temperature were controlled by the Langevin piston method [41]. The atomic coordinates were saved every 2.0 ps and thus 10000 structures were collected in each system for further analysis.

### Principal Component Analysis

Principal component analysis (PCA) can provide a brief picture of motions, which exacts the highly correlated fluctuations from the MD trajectories by applying the dimensionality reduction method. This method is based on the calculation and diagonalization of the covariance matrix. The elements 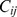 in the matrix are defined by [42]:
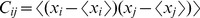
(1)where 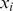
(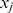) is the coordinate of the 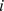
th(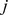
th) atom of the systems, and 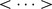 indicates an ensemble average. The eigenvectors (also called the principal modes) of the matrix represent the directions of the concerted motions and the eigenvalues indicate the magnitude of the motions along the direction. Usually, the first few principal components (PCs) describe the most important slow modes of the system, which are related to the functional motions of a biomolecular system [43], [44]. In this article, PCA was performed with Gromacs 4.5 package [45] in order to investigate and compare the functional motions of DNA-free and DNA-bound dHax3.

### Free Energy Landscape

Free-energy landscape (FEL) can promote our understanding of biomolecular processes such as molecular recognition, folding and aggregation [42], [44]. The free-energy minima represent the conformational ensemble in stable states which are accessible to a biomolecule under physiological conditions. And the free-energy barriers denote the transient states connecting them. The FEL can be constructed on the basis of PCA [42]. The corresponding expression is
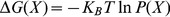
(2)where 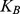 and 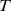 are the Boltzmann constant and absolute temperature, 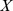 stands for the PCs and thus 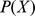 is the probability distribution of the molecular system along the PCs. In our study, we calculated the FEL to identify the dominant conformational states with relatively lower energies.

### Conformational Analysis of Nucleic Acids

Curves is a widely used nucleic acid conformational analysis program [46]. The program provides a full analysis of DNA structure, including base pair-axis parameters, intra-base and inter-base pair parameters, backbone and groove parameters, etc. In our study, 1000 snapshots were extracted from 20 ns dynamics by sampling every 20 ps. The following parameters were analyzed to describe the DNA structural deformability, including axis bend angles, slides, roll angles, twist angles, rises and groove widths.

## Results and Discussion

### Convergence Behavior of the Two MD Simulations

**Figure 2:**
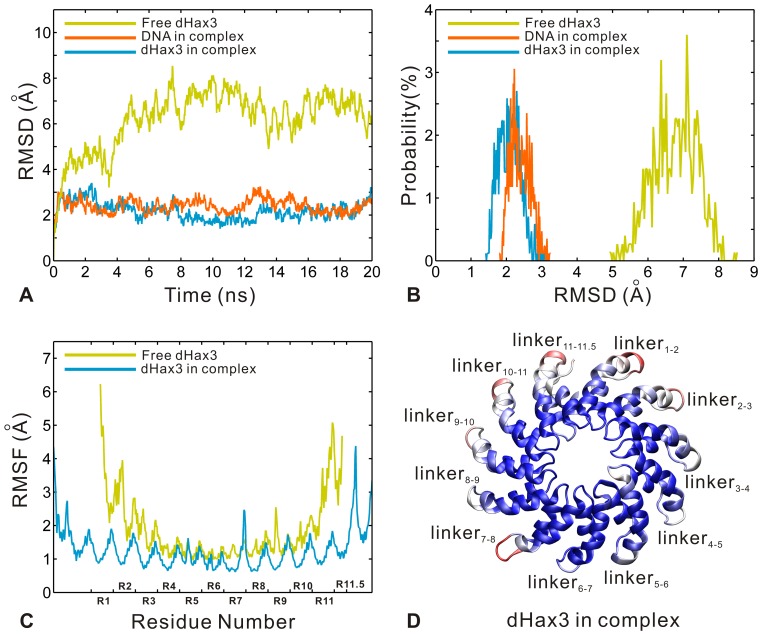
Comparative MD analysis of DNA-free dHax3 (yellow) and DNA-bound dHax3 (dHax3: sky blue; DNA: orange) systems. (A) The RMSD values of the dHax3 and DNA backbone atoms versus simulation time. (B) The probability distribution of RMSD calculated from the equilibrium trajectories. (C) The RMSF values of the dHax3 Cα atoms calculated from the equilibrium trajectories. (D) The cartoon representation of 11.5 TAL repeats for the dHax3 in the DNA-bound system. The residues in red have relatively higher RMSF values (>1.8 Å) while the ones in blue have relatively lower RMSF values (<1 Å). The other regions are colored white.

Through 20 ns MD simulations, the systems reached equilibrium by checking the evolutions of potential energies, temperatures and volumes versus time (see Figure S1). The root-mean-square deviation values (RMSDs) were calculated over the dHax3 and DNA backbone atoms relative to the initial structures, and the results are shown in Figure 2 A. The last 15 ns and 17 ns MD trajectories remain comparatively stable and then are taken as the equilibrium portions for the DNA-free and DNA-bound systems, respectively. In view of previous MD studies of protein-DNA [47], [48], this simulation protocol is proper to describe the two systems. Figure 2 B displays the distributional probability of RMSD from the equilibrium trajectories in the two systems. The RMSDs converge to about 2.09 Å, 2.39 Å, and 6.76 Å for the dHax3 in complex, the DNA in complex and the free dHax3, respectively. The RMSDs of the DNA-bound system are significantly lower than those of the DNA-free system. It indicates that the TALE dHax3 is well constrained in the DNA-bound system. The conformational changes along the simulation trajectories are shown in Movie S1 and S2 for the DNA-free and DNA-bound systems, respectively. The detailed mechanism of dHax3 and DNA will be analyzed in the following sections.

Furthermore, we also calculated the root-mean-square fluctuation (RMSF) values for Cα atoms of dHax3 from the equilibrium trajectories in the two systems. The result is shown in Figure 2 C, where 11.5 TAL repeats are labeled as R1 to R11.5, respectively. The comparison of RMSF values reveals the markedly reduced fluctuations of the dHax3 in complex (sky blue) relative to the DNA-free dHax3 (yellow). From DNA-free to DNA-bound states, the fluctuation decrease of dHax3 shows two important characteristics. One is that the RVD loop region has less fluctuation than other parts of the repeat. The other is that the two ends of tandem repeats contribute most to the reduction in the fluctuations. Previous studies [19], [20], [29] described that the RVD loop regions in TAL repeats are the DNA binding sites. In the DNA-bound system, almost all RVD loop regions show decreased RMSF values while all linkers between two adjacent TAL repeats still maintain relatively higher RMSF values (see Figure 2 C). The fluctuation decrease in the RVD loop regions implies the importance of the specific binding of TALE-DNA to the system stability. Meanwhile, more remarkable decreases of RMSF values are found in the two ends than the middle of tandem repeats (see Figure 2 C). It indicates that the TALE-DNA binding of the two ends has more contributions to the system stability than that of the middle. In the structure of dHax3, most of the repeats with RVDs HD locate at the ends of the 11.5-repeat effector (see Figure 1 B). RVDs HD were considered to be efficient for the specific recognition of DNA sequence, which are important to the overall TALE activity [24]. Thus, the contribution to the system stability may be associated with the RVD efficiency of the specific recognition. Notably, the linker between repeat 7 and repeat 8 (abbreviated as linker_7–8_) exhibits an increase in RMSF values in the DNA-bound system compared with the DNA-free system (see Figure 2 C). In order to intuitively observe the fluctuations in the DNA-bound system, 11.5 TAL repeats of the dHax3 are colored according to the RMSF values. The regions with high RMSF values are shown in red (see Figure 2 D). In the crystal structure, the linker_7–8_ exhibits a slightly loose structure and the other linkers display a relatively more regular helix conformation. The conformational difference leads to the higher RMSF values of the linker_7–8_. The rest of red regions locate at the linker_1–2_, linker_2–3_, linker_10–11_ and linker_11–11.5_. Therefore, it is speculated that the dHax3 may possess an important conformational change at the ends of the superhelical structure.

### Interactions between the dHax3 and the DNA

The crystallographic study [19] revealed that there are both direct and water-mediated hydrogen bonds in the DNA-bound dHax3 structure. They mediate the important interactions in the TALE-DNA binding. Furthermore, the direct hydrogen bonds can be classified into two types: the specific interaction between amino acid and DNA base; and the nonspecific interaction between amino acid and DNA backbone [49]. Then, we examined the hydrogen bonds between 11.5 TAL repeats and the DNA from the equilibrium trajectory of the DNA-bound system. The hydrogen bonds were calculated by VMD 1.9 [36] with a distance cut-off value of 3.5 Å and an angle cut-off value of 35°. The results are listed in Table 1 (direct hydrogen bonds) and Table 2 (water-mediated hydrogen bonds) with occupancy over 40%. The atom OD1 (OD2) of the residue ASP13 in repeats 1, 2 and 9 (containing RVD HD) accepts a hydrogen bond from the atom N4 of base C. The atom OG of the residue Ser13 in repeat 7 (containing RVD NS) also donates a hydrogen bond to the atom N7 of base A (see Table 1). These base-specific hydrogen bonds are important for the recognition of base C by RVD HD and the recognition of base A by RVD NS [50]. The base T is usually recognized by RVD NG [3], [19], [20], however, no base-specific hydrogen bond is found between bases T and residues Gly13 in all repeats with RVDs NG. Relative to the base C, the base T needs sufficient space to accommodate its 5-methyl group. Due to the lack of side chain in glycine, Gly13 can provide enough space to the base T and make a van der Waals contact with the 5-methyl group of base T [19]. In contrast, any other residues with a side chain at position 13 may introduce steric clash with the base T. Thus, the van der Waals interaction plays a key role in the recognition of base T by RVD NG. Consequently, the efficiency of HD (strong RVD) is considered to be higher than that of NG (weak RVD) [24]. In addition, the residues at position 13, 14, 16 and 17 in the repeats are involved in the phosphate binding with the DNA backbone (see Table 1 and 2). The 17th residue forms direct hydrogen bonds with the phosphate group of DNA. The 13th, 14th and 16th residues form water-mediated hydrogen bonds with the phosphate group of DNA. These nonspecific hydrogen bonds are helpful to the structural stability of the DNA-bound system. The above observed interactions are in agreement with experimental data [19]. Interestingly, although repeat 11.5 is a truncated repeat with only containing the first 20 residues, the half repeat still forms relatively stronger nonspecific interactions with the DNA backbone (see Table 1 and 2). It is suggested that the last half repeat of TALE makes an important contribution to the system stability.

**Table 1:** TALE-DNA direct hydrogen bonds in 11.5 repeats with occupancy over 40%.

Repeat	TALE (Residue id*)	Sense-strand DNA base	Occupancy
1	**D301-OD2 (13)**	**C1-N4**	**47.76%**
2	**D335-OD1 (13)**	**C2-N4**	**49.88%**
4	Q407-NE2 (17)	C3-O1P	79.65%
6	Q475-NE2 (17)	T5-O1P	52.35%
7	**S505-OG (13)**	**A7-N7**	**66.59%**
	Q509-NE2 (17)	T6-O1P	72.59%
8	Q543-NE2 (17)	A7-O2P	58.82%
9	**D573-OD1 (13)**	**C9-N4**	**50.94%**
	Q577-NE2 (17)	T8-O1P	74.35%
10	Q611-NE2 (17)	C9-O1P	62.12%
11	Q645-NE2 (17)	T10-O1P	84.59%
11.5	R678-NE (16)	C11-O1P	90.24%
	R678-NH2 (16)	C11-O1P	42.82%

Residue id* is the index of a residue in each TAL repeat sequence. The specific interactions are in bold while non-bold denotes nonspecific interactions.

**Table 2:** Water-mediated hydrogen bonds in 11.5 repeats with occupancy over 40%.

Repeat	TALE (Residue id*)	Sense-strand DNA base	Occupancy
2	G336_O (14)	C2_O2P	53.18%
3	K372_N (16)	C2_O2P	89.29%
	D369_O (13)	C3_O2P	78.24%
	G370_N (14)	C2_O2P	53.06%
	D369_OD1 (13)	T4_O4	47.65%
4	K406_N (16)	C3_O2P	92.94%
	G403_O (13)	T4_O2P	54.24%
5	K440_N (16)	T4_O2P	55.41%
7	G506_N (14)	T6_O2P	73.53%
	S505_O (13)	A7_O1P	50.35%
	K508_N (16)	T6_O2P	57.06%
8	K542_N (16)	A7_O1P	90.82%
	G539_O (13)	T8_O2P	74.47%
	G540_N (14)	A7_O1P	44.00%
9	K576_N (16)	T8_O2P	93.53%
	D573_O (13)	C9_O2P	73.41%
	G574_N (14)	T8_O2P	66.71%
10	K610_N (16)	C9_O2P	88.24%
	G607_O (13)	T10_O2P	70.24%
11	K644_N (16)	T10_O2P	91.29%
	D641_O (13)	C11_O2P	70.24%
11.5	R678_N (16)	C11_O2P	93.18%
	G676_O (14)	T12_O2P	83.18%

Residue id* is the index of a residue in each TAL repeat sequence.

Previous study [24] revealed that tandem weak repeats (containing RVDs NG or NI) can compromise TALE function. In this study, RVDs NG locate in repeats 4, 5, 6, 8, 10 and 11.5. According to the above analysis, Gly13 in repeats with RVDs NG forms a van der Waals interaction with the corresponding base T. It is required for the recognition of base T by RVD NG. Thus, we investigated the distance between the Cα of Gly13 and the 5-methyl group of base T for each repeat with RVD NG. Table 3 provides the distance data between them in repeats 4, 5, 6, 8, 10 and 11.5, including initial distances, average distances and distance deviations. The distances of repeats 5 and 6 increase remarkably than those of other repeats. It implies that the repeats 5 and 6 have weak van der Waals interactions relative to repeats 4, 8, 10 and 11.5. Meanwhile, repeats 4, 8, 10 and 11.5 form three or four hydrogen bonds with the phosphate group of bases C3, A7, C9 and C11, respectively (see Table 1 and 2). In comparison, repeats 5 and 6 only have one hydrogen bond with the phosphate group of bases T4 and T5, respectively. Collectively, the TALE-DNA interactions in repeats 5 and 6 are weaker than those in repeats 4, 8, 10 and 11.5. As shown in Figure 1 C, the predecessors of repeats 4, 8, 10 and 11.5 contain RVDs HD or NS while those of repeats 5 and 6 still include RVDs NG. Therefore, tandem weak RVDs (like NG) are unfavorable for the association between TALE and DNA. Our study supports the recommendation of avoiding stretches of weak RVDs in TALE design [24].

**Table 3:** Distances between the Cα of G13 and the 5-methyl group of thymine in all repeats with RVDs NG.

Repeat	D_Initial_ a	D_Average_ b	ΔD^c^
4	3.68	3.98	0.30
5	3.64	4.88	1.24
6	5.26	7.93	2.67
8	3.79	3.85	0.06
10	3.61	3.98	0.37
11.5	3.88	4.12	0.24

All distances are measured in the unit of Å.

a D_Initial_ is the distance from the crystal structure.

b D_Average_ is the mean value of distances from the equilibrium trajectory.

c ΔD describes the deviation between D_Initial_ and D_Average_.

**Figure 3:**
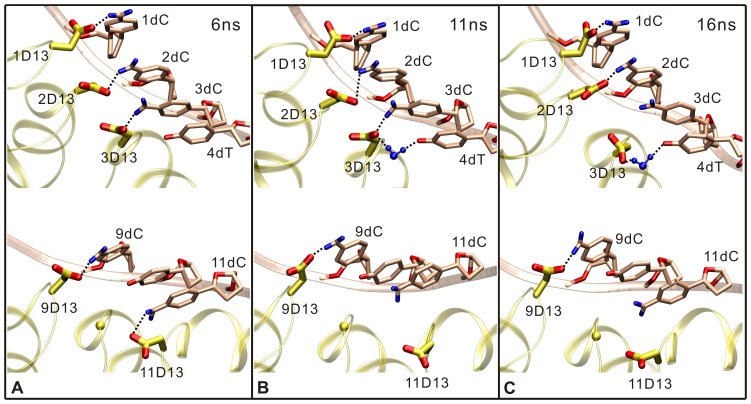
The change of the specific hydrogen bonds between the TAL repeats and DNA bases at 6(A), 11 ns (B) and 16 ns (C) in the DNA-bound system. The TAL repeats (yellow), DNA (pink) and water (blue) are depicted with ribbons, tube and CPK models, respectively. During the simulation, 1D13 (the residue Asp13 in repeat 1), 2D13, and 9D13 kept the specific hydrogen bond with 1dC (the base C1 in the DNA target sequence), 2dC, and 9dC, respectively. However, 11D13 lost the specific hydrogen bond with 11dC after 6 ns. Interestingly, 3D13 firstly interacted with 3dC (A). At 11 ns, 3D13 also formed water-mediated hydrogen bond with 4dT (B). Finally, 3D13 only indirectly contacted with 4dT (C).

Notably, the interactions between the repeats containing RVDs HD and the DNA are found to vary according to the different positions in the effector. Figure 3 describes the change of specific interactions between the DNA bases and the residues Asp13 in the repeats containing RVDs HD (1, 2, 3, 9 and 11). Repeats 1 and 2, as the beginning of tandem repeats, form the specific interactions with DNA bases C1 and C2, respectively. However, they have less phosphate interactions with the DNA backbone, especially none for repeat 1 (see Table 1 and 2). The residue Asp13 in repeat 3, rather than directly interacting with the atom N4 of base C3 (with occupancy 26.82%), prefers to form a water-mediated hydrogen bond with the atom O4 of base T4 (with occupancy 47.65%). Repeat 9, that locates relatively near the middle of tandem repeats, forms a base-specific hydrogen bond with base C9. Meanwhile, it also maintains the stable phosphate binding with the DNA (see Table 1 and 2). In contrast to repeat 1, repeat 11 at the end of tandem repeats loses the specific hydrogen bond with base C11 (see Figure 3 B and C) and only has the stable phosphate binding with the DNA (see Table 1 and 2). The changes of interactions occur frequently at the head and tail of tandem repeats, which may be related to the functional motions of TALE dHax3.

### Slow Modes of the Motions

**Figure 4:**
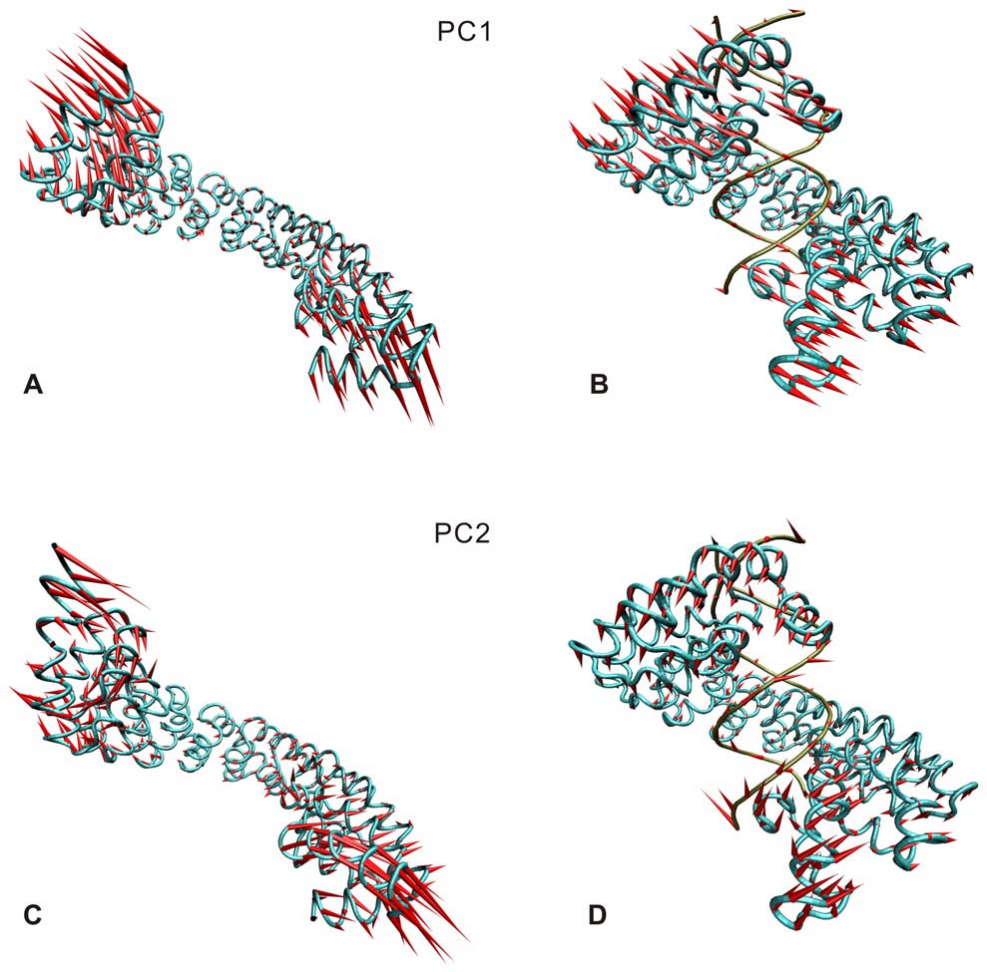
The first and second slowest motion modes of the DNA-free system (A and C) and DNA-bound system (B and D). The length of cone is positively-correlated with motive magnitude, and the orientation of cone indicates motive direction. The two systems have the similar slow motions. The first slowest motion mainly appears as the open-close movements between the two ends of the superhelical structure (A and B). The second slowest motion shows a twisting around each end (C and D).

In order to inspect the functional motions of superhelical structure, the PCA analysis was performed for Cα atoms of 11.5 repeats and P atoms of DNA based on the equilibrium trajectories. Figure 4 compares the first and second slowest motion modes of the DNA-free and DNA-bound systems. Similar slow modes are evident in the two systems: the first slowest motion mainly appears as the open-close movements between the two ends of the superhelical structure (see Figure 4 A and B); the second slowest motion shows a twisting around each end (see Figure 4 C and D). In view of the similar motion modes in the DNA-free and DNA-bound systems, the slow motions of two ends are likely to be the intrinsic property of dHax3. It may be associated with TALE function. In addition, the DNA also shows similar but weakened motion trend to the dHax3 in the DNA-bound system.

**Figure 5:**
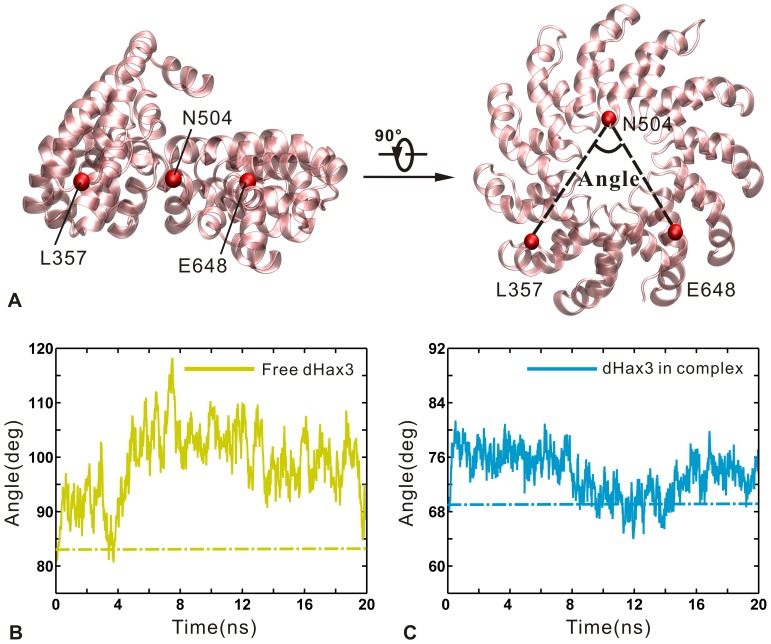
Representation of the intramolecular angle and changes of the angles in the DNA-free and DNA-bound systems. (A) The cartoon representation of 11.5 TAL repeats, with the intramolecular angle being defined by the Cα atoms of L357 (Leu357), N504 (Asn504) and E648 (Glu648). (B) The intramolecular angle change versus simulation time (solid line) and the value from crystal structure (dotted line) for the DNA-free dHax3. (C) The intramolecular angle change versus simulation time (solid line) and the value from crystal structure (dotted line) for the DNA-bound dHax3.

For evaluating the open-close movements in the first slowest motion mode, we define an intramolecular angle to measure the conformational changes. The angle is formed by the three atoms: the Cα atoms of Leu357 (repeat 3), Asn504 (repeat 7) and Glu648 (repeat 11), which are selected from the beginning, middle and end of the superhelical structure, respectively (see Figure 5 A). In the DNA-free system, the angle increases from the initial value about 81° to the maximum value about 115° before 7 ns. Then, the angle rapidly drops down to 100°. After 8 ns, it shows the slightly decreasing trend and fluctuates around 97° (see Figure 5 B). In the DNA-bound system, the angle keeps stable about 76° before 8 ns. From 8∼15 ns, the angle markedly decreases to 64° and then rises back again. After 15 ns, the angle remains about 74° until the end of the simulation (see Figure 5 C). Due to the constraints from the DNA, the angle of DNA-bound dHax3 shows a lower value and a narrower fluctuation range than that of the DNA-free dHax3. Notably, in the two systems the values of intramolecular angle show a positive correlation with the RMSDs of the dHax3 backbone atoms during 20 ns simulation time. Specially, their correlation coefficients are 0.79 for the DNA-free system and 0.64 for the DNA-bound system (see Figure S2 A and B). It indicates that the open-close movements have a major influence on the system stability.

**Figure 6:**
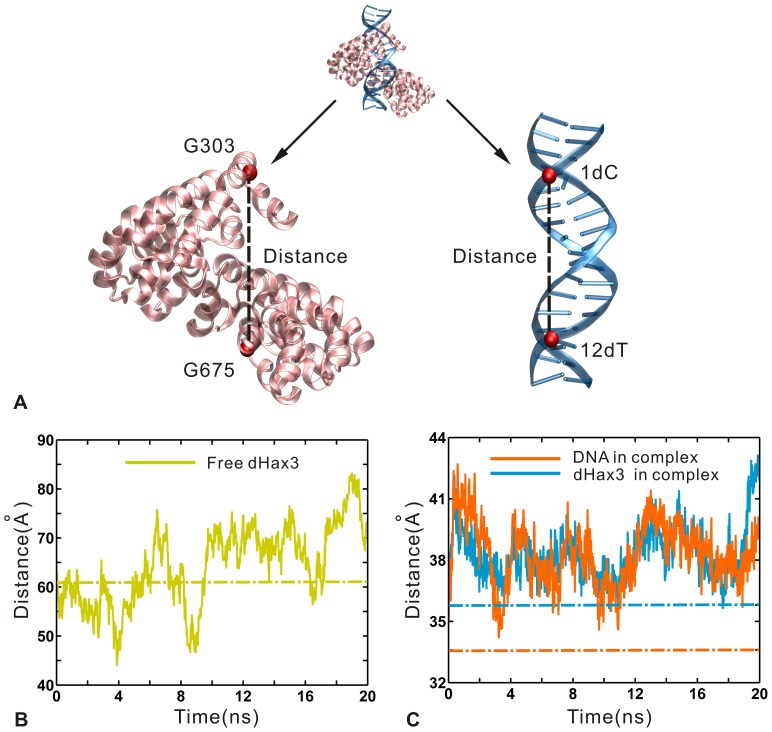
Representation of the superhelical pitches and changes of the distances in the DNA-free and DNA-bound systems. (A) The cartoon representation of 11.5 TAL repeats and the DNA. The superhelical pitch of dHax3 is assessed by the distance between the Cα atoms of G303 (Gly303) and G675 (Gly675) (left), and the pitch of DNA by the distance between the C3′ atoms of 1dC and 12dT (right). (B) The distance change versus simulation time (solid line) and the value from crystal structure (dotted line) in the DNA-free system. (C) The distance change versus simulation time (solid line) and the value from crystal structure (dotted line) in the DNA-bound system.

The crystallographic study [19] showed a remarkable difference between the superhelical pitch of DNA-free dHax3 (about 60 Å) and that of DNA-bound dHax3 (about 35 Å). During the simulation, the slow motions are also found to cause the visible change of superhelical pitch. In this study, the pitch change is assessed by the distance between the Cα atoms of Gly303 (repeat 1) and Gly675 (repeat 11.5) for the dHax3, and by the distance between the C3′ atoms of C1 and T12 for the DNA, respectively (see Figure 6 A). In the DNA-free system, the distance fluctuates between 45 Å and 80 Å (see Figure 6 B). In the DNA-bound system, the dHax3 and DNA have the similar distance fluctuation range from 35 Å to 43 Å (see Figure 6 C). On one hand, relative to the DNA-free dHax3, the DNA-bound dHax3 is remarkably compressed by the constraint from the DNA. On the other hand, the DNA is also observed to be pulled by the DNA-bound dHax3 in comparison with the crystal value (see Figure 6 C). Thus, the distance of DNA changes along with that of the dHax3 in the DNA-bound system. Notably, the large fluctuations in distance correspond to the frequent changes of the superhelical pitch. It exhibits a dramatic conformational plasticity of effector, which is likely important for the function of TALEs [18], [19].

### Functional Conformation Changes of dHax3

**Figure 7:**
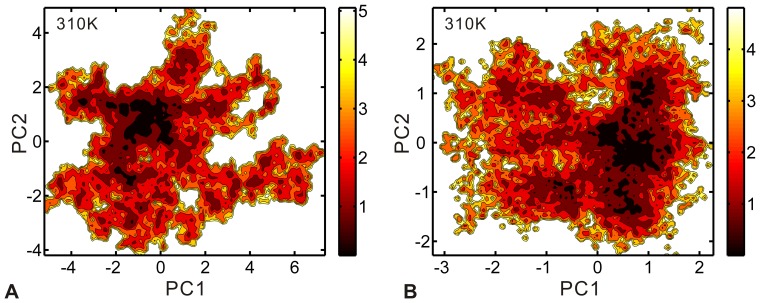
Free energy contour map versus the principal components PC1 and PC2 at 310-free system (A) and DNA-bound system (B). Deeper color indicates lower energy.

The PCA analysis reveals that the slow motions lead to remarkable conformational changes of dHax3. Then, we further investigated the distribution of conformations along the PCs. Figure 7 displays the free energy contour maps of the two systems at 310 K, with deeper color indicating lower energy. In the DNA-free system, the local minima approximately in the upper left of the FEL (see Figure 7 A). The minima mainly correspond to the conformations from 9∼13 ns and 16∼18 ns (see Figure S3 A). Relative to the DNA-free system, there are more local minima in the DNA-bound system (see Figure 7 B). They are almost in the right of the FEL, of which the largest one lies in the lower right. These minima include the conformations from 4∼8 ns and 15∼20 ns (see Figure S3 B). In the two systems, these stable conformations are almost characterized by a relatively stable intramolecular angle (see Figure 5 B and C). In the DNA-free system, the conformations in the local basin have an intramolecular angle around 100°. In the DNA-bound system, the intramolecular angle values of the local basin are about 75°. It is consistent with the important influence of open-close movements on the system stability in the above PCA analysis.

Compared with the crystal values (DNA-free: 69.1°; DNA-bound: 83.2°), the dHax3 in the two systems is more stable with a more open intramolecular angle when it is relaxed in solvent environment. Meanwhile, there is an important open-close conformational change between the DNA-free and DNA-bound dHax3 systems. The intramolecular angle of the DNA-free dHax3 is always higher than 80° and that of the DNA-bound dHax3 is almost lower than 80°. The conformational plasticity of TALE has been considered to be important for TALE function [18], [19]. Compared with the DNA-bound TALE, the DNA-free TALE presents a relatively more extended and unwound conformation [18], [19], which was suggested to be required for a DNA target search by the unbound TALE [51]. We further speculate that a more open intramolecular angle higher than 80° (for example 100°) is necessary for the DNA target search in the DNA-free TALE. After binding with the target sequence of DNA, the TALE needs to form close contacts with the DNA. Then, it decreases the intramolecular angle to less than 80° (for example 75°). The conformational change of dHax3 is induced by the DNA binding, which is suggested to be an essential step in the TALE-DNA recognition.

### Structural Deformability of DNA

**Figure 8:**
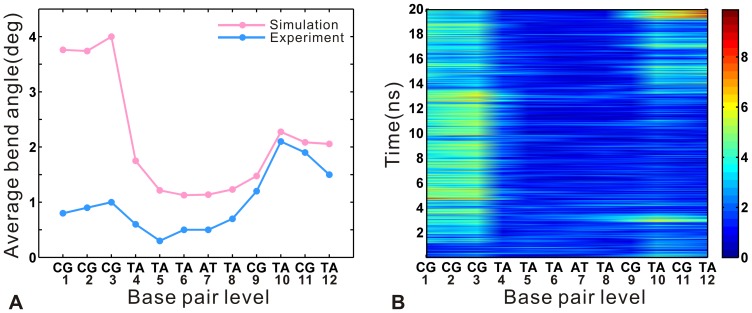
Changes of axis bend angles along the DNA target sequence. (A) Comparison of the average values (pink) of the axis bend calculated from the equilibrium trajectory along the d(CCCTTTATCTCT) with the corresponding crystal values (sky blue). (B) Fluctuations of axis bend for all dinucleotide steps along the d(CCCTTTATCTCT) during 20 ns trajectory. The color bar gives the variations in bend from 0° (dark blue) to 10° (dark red).

**Figure 9:**
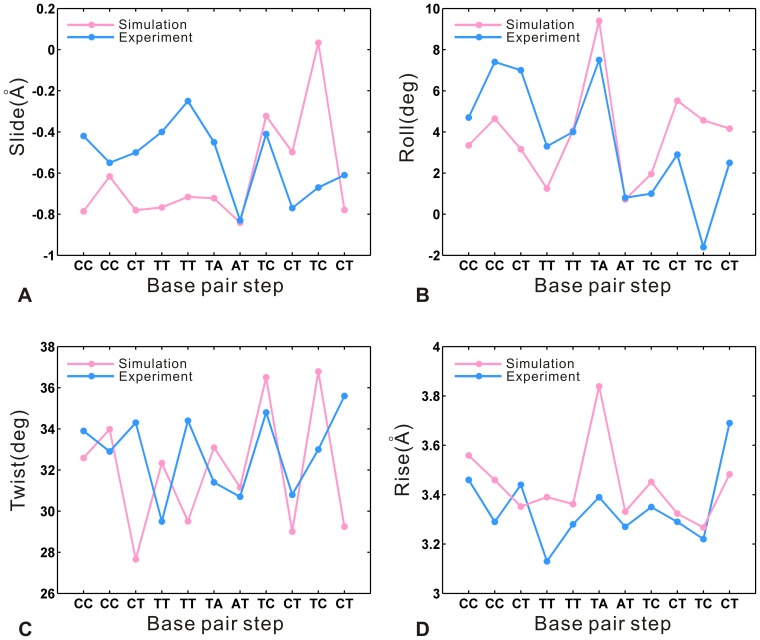
Comparison of the average values (pink) of base pair step parameters calculated from the equilibrium trajectory along the d(CCCTTTATCTCT) with the corresponding crystal values (sky blue). (A) Slide. (B) Roll angle. (C) Twist angle. (D) Rise.

The deformability of DNA plays an important role in the biological processes [32]–[35]. Then, it is necessary to analyze the flexibility of DNA at the base-pair level. The DNA structural parameters are calculated for the DNA target sequence (C1-C2-C3-T4-T5-T6-A7-T8-C9-T10-C11-T12). The previous study [52] indicated that DNA bending is an important structural feature in protein-DNA recognition. Figure 8 A compares the mean bend angle from the equilibrium trajectory with the crystal values along the DNA target sequence. The mean angle shows an increase at all target sites, especially remarkable at the 5′ end (C1-C2-C3). Figure 8 B describes the time evolution (on the vertical axis) of the axis bends (on the horizontal axis) at all base pair steps. During the last 6 ns, the bend angle values of the sites at the 5′ end and 3′ end (T10-C11-T12) are higher than those of the middle sites along the target sequence. It indicates the two ends have a higher bending degree relative to the middle parts. Meanwhile, the DNA bending is associated with the roll and slide of inter-base-pair, for example, the negative values of slide appear with DNA bending into the major groove where roll is positive [53]. Then, Figure 9 compares the average DNA structural step parameters from the equilibrium trajectory with the crystal values, including slide, roll angle, twist angle and rise. As shown in Figure 9 A and B, the bending of DNA is accompanied by the negative slide and positive roll angles. Thus, the DNA sequence shows the increased major groove bending, with higher bend angle values at the ends (see Figure 8 B). The comparison of twist angle is presented in Figure 9 C, where the simulated result shows the decreasing tendency with the average value of 32° relative to 33° in the crystal structure. The previous study [53] indicated that the double helix tends to overwind at the sites of minor groove bending and to underwind at the sites of major groove bending. Meanwhile, overwinding of the helix increases the twist angle and underwinding decreases it [54]. Thus, the reduced twist is associated with the increased major groove bending degree, especially at the sites of the first half of DNA target sequence. Moreover, as shown in Figure 9 D, the rise from the simulated result increases compared with the crystal values. It corresponds to the growth of the distance between the two ends of the DNA target sequence (see Figure 6 C). Altogether, compared with the other positions along the target sequence, the 5′ end is significantly distorted with a more remarkable increase of bend angle and higher bend angle values. The observation suggests that the efficiency of specific recognition is higher at the 5′ end than the other sites along the target sequence.

**Figure 10:**
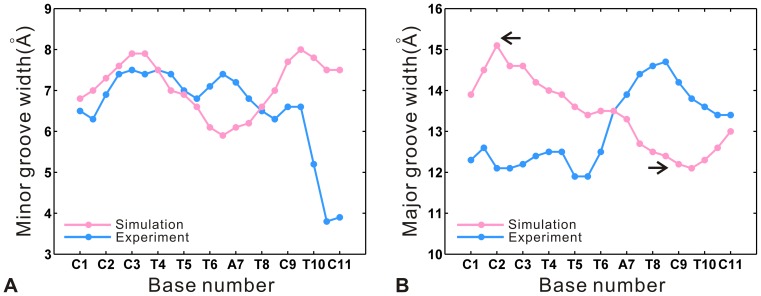
Comparison of the average values (pink) of groove widths calculated from the equilibrium trajectory along the d(CCCTTTATCTCT) with the corresponding crystal values (sky blue). (A) Minor groove widths. (B) Major groove widths. The widening of the major groove is more remarkable at the sites of C2 and C9.

The width of DNA grooves is also an important parameter of DNA structure, which is often correlated with protein-DNA interactions [55], [56]. Figure 10 A and B display the comparison of the average groove widths from the equilibrium trajectory with the crystal values. There are remarkable variations in groove widths at the ends along the target sequence, including the increase of major groove widths at the 5′ end and that of minor groove widths at the 3′ end (see Figure 4 B). The major groove is widened by about 3 Å at the sites of C1-C2-C3 (see Figure 10 B), and the minor groove widths are also increased by about 3.5 Å at the sites of bases C9-T10-C11 (see Figure 10 A). Meanwhile, an opening of the minor groove is often associated with the compression of the major groove [46], [57]. Thus, a decrease of about 2.5 Å in major groove widths occurs at the sites of T8-C9-T10 (see Figure 10 B), which corresponds to the increase of about 3.5 Å in the minor groove widths at the sites of C9-T10-C11. Previous studies [58], [59] indicated that minor groove is narrower in AT-rich central regions (of four or more successive AT base pairs) compared to GC-rich regions. Consequently, our result indicates that the minor groove is compressed by 1.5 Å at the central site of T4-T5-T6-A7-T8 segment relative to the crystal values. Notably, the bases C2 and C9 have more remarkable widths variations in the major groove (see Figure 10 B). By comparing the hydrogen bonds at different sites along the target sequence, it is found that C2 and C9 form relatively stronger specific interactions of TALE-DNA (see Table 1). Although the base A7 also forms one high occupancy hydrogen bond with the residue Ser13 in repeat 7, the interaction previously was designated as nonspecific because NS is nonselective to recognize all four bases [6]. Then, the specific interaction of TALE-DNA is likely favorable for the variations of major groove widths. Further, the sites at the 5′ end of DNA target sequence form more specific interactions than those at the 3′ end (see Figure 3). Correspondingly, the 5′ end shows higher bend angle and more variations in the major groove than the 3′ end (see Figure 8 A and 10 B). It is suggested that the conformational change of DNA is associated with the specific interaction of TALE-DNA. Meanwhile, the 5′ end has more remarkable structural deformability relative to the 3′ end, which also indicates that the N-terminal repeats have more contributions to the specific recognition of TALE-DNA than the C-terminal ones. It is consistent with the previous experimental study [25] that N-terminal repeats are more important to the overall affinity than C-terminal ones. Therefore, we suppose that the arrangement of N-terminal repeats with strong RVDs (that can form specific hydrogen bond with DNA bases, like HD) may be helpful to the efficient design of customized TALEs.

## Conclusions

In this study, MD simulations were performed to investigate the TALE-DNA recognition mechanism. The simulated results indicate that the fluctuations of DNA-bound dHax3 are reduced significantly relative to the DNA-free dHax3. It results from the specific binding between RVDs (repeat-variable diresidues) in the repeats and the DNA. Meanwhile, the N-terminal and C-terminal repeats contribute most to the decreased fluctuations. By calculation of specific and nonspecific hydrogen bonds at the interface, it is found that within a TAL repeat the residue at position 13 forms a specific interaction with the corresponding DNA base, and the residues at position 13, 14, 16 and 17 are important to the phosphate binding with the DNA backbone. The observed interactions in our study are in good agreement with experimental data. The last half repeat of TALE has an important contribution to the nonspecific interactions with the DNA backbone. It suggests that the last half repeat of TALE helps to stabilize the TALE-DNA complex. Moreover, tandem repeats with weak RVDs are shown to be unfavorable for the interaction of TALE-DNA. It is consistent with the previous suggestion of avoiding stretches of weak RVDs in the design of TALEs. The PCA analysis reveals similar slow modes of motions in both DNA-free and DNA-bound systems. The dominant motions of the two systems are both open-close movements between the two ends of the superhelical structure. The movements lead to the changes of the intramolecular angle. It is suggested to be an essential step in the TALE-DNA recognition. By comparing DNA structural parameters, the 5′ end of the target sequence has more remarkable increases of bend angle and variations in major groove widths relative to the 3′ end. It reveals the importance of N-terminal repeats for the specific recognition of TALE-DNA. Meanwhile, the conformational change of DNA is likely related to the specific interaction of TALE-DNA. Therefore, we suppose that the arrangement of N-terminal repeats with strong RVDs may help to construct functional TALEs. This study provides a deeper understanding to the recognition mechanism of TALE-DNA, and may facilitate the TALE design as artificial gene targeting reagents for biotechnological applications.

## Supporting Information

Figure S1 **The energies (A and B), temperatures (C) and volumes (D) versus simulation time in the two systems.** All the energies of potential (blue), kinetic (red) and total ( = potential+kinetic, black) in MD simulations for the DNA-free (A) and DNA-bound (B) systems, respectively. The temperatures and volumes of the DNA-free (orange) and DNA-bound (green) systems, are also given in (C) and (D). The plots of energies, temperatures and volumes level off. It indicates that the equilibriums are reached.
(TIF)

Figure S2 **Correlations between the values of intramolecular angle and the RMSDs of the backbone atoms for the DNA-free (A) and DNA-bound (B) dHax3.** The correlation coefficients are 0.79 for the DNA-free dHax3 and 0.64 for the DNA-bound dHax3, respectively.
(TIF)

Figure S3 **The principal components PC1 and PC2 versus simulation time in the DNA-free (A) and DNA-bound (B) systems.** For the free-energy minima in the DNA-free system, the values of PC1 vary from −3.8 to 0.8 and those of PC2 from −0.3 to 1.6 (see Figure 7 A). For the free-energy minima in the DNA-bound system, the values of PC1 vary from 0 to 1.5 and those of PC2 from −1.5 to 1.3 (see Figure 7 B). Then, the time sections can be determined by searching the corresponding intervals of PC1 and PC2 (dotted line). Approximately, the free-energy minima in the DNA-free system (yellow) correspond to the segments of 9∼13 ns and 16∼18 ns, while those in the DNA-bound system (sky blue) correspond to the segments of 4∼8 ns and 15∼20 ns.
(TIF)

Movie S1 **This shows the conformational changes of 11.5 TAL repeats along the simulation trajectory in the DNA-free system.**
(MPG)

Movie S2 **This shows the conformational changes of 11.5 TAL repeats and DNA along the simulation trajectory in the DNA-bound system.**
(MPG)
