# Supplementary figures and images for: Molecular Dynamics Simulations of DNA-Free and DNA-Bound TAL Effectors
Source: PLoS One. 2013 Oct 10;8(10):e76045. doi: 10.1371/journal.pone.0076045 (PMC3794935; doi:10.1371/journal.pone.0076045)

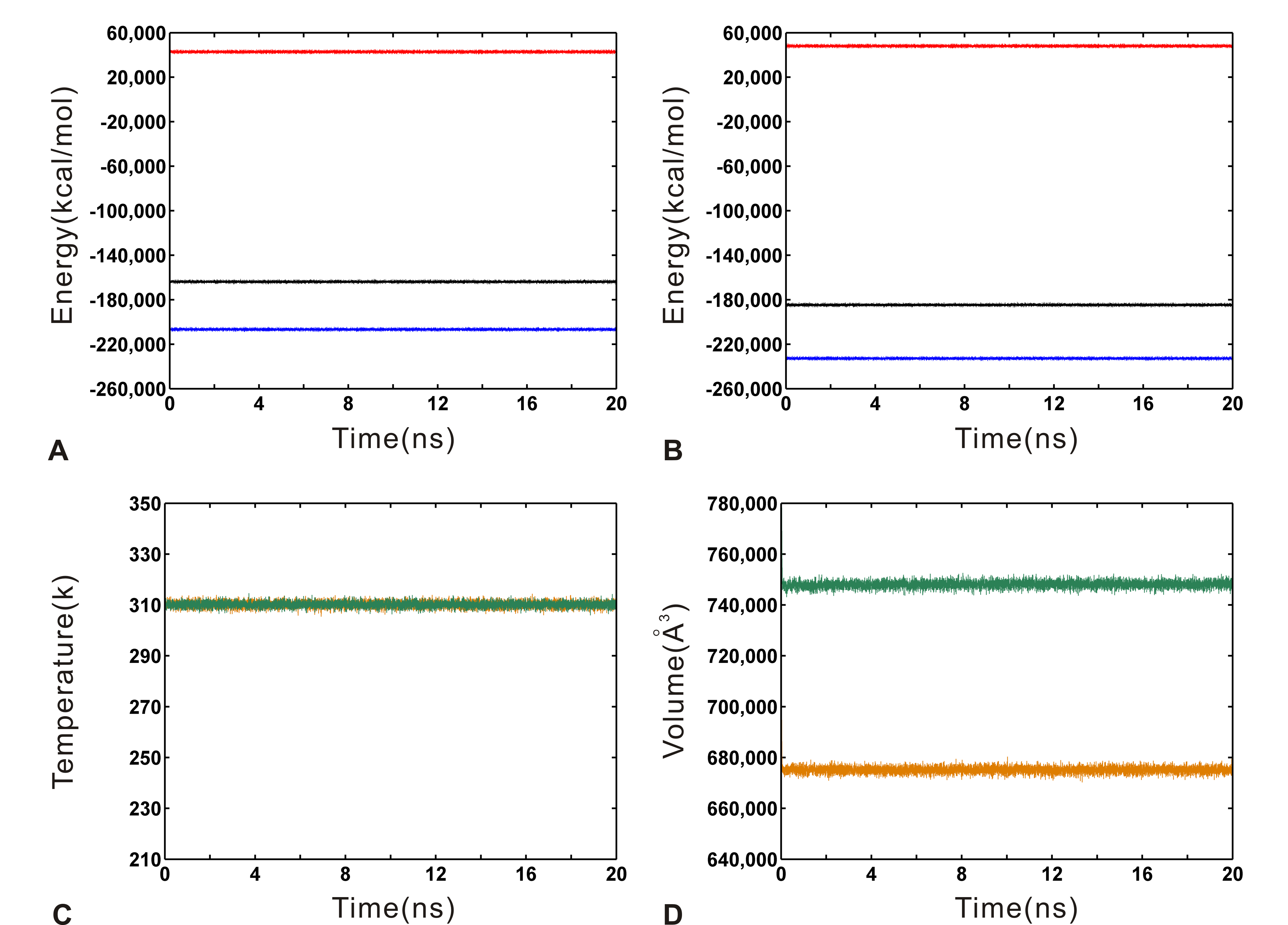

Supplement: Figure S1 — The energies (A and B), temperatures (C) and volumes (D) versus simulation time in the two systems. All the energies of potential (blue), kinetic (red) and total ( = potential+kinetic, black) in MD simulations for the DNA-free (A) and DNA-bound (B) systems, respectively. The temperatures and volumes of the DNA-free (orange) and DNA-bound (green) systems, are also given in (C) and (D). The plots of energies, temperatures and volumes level off. It indicates that the equilibriums are reached. (TIF) [file pone.0076045.s001.tif]

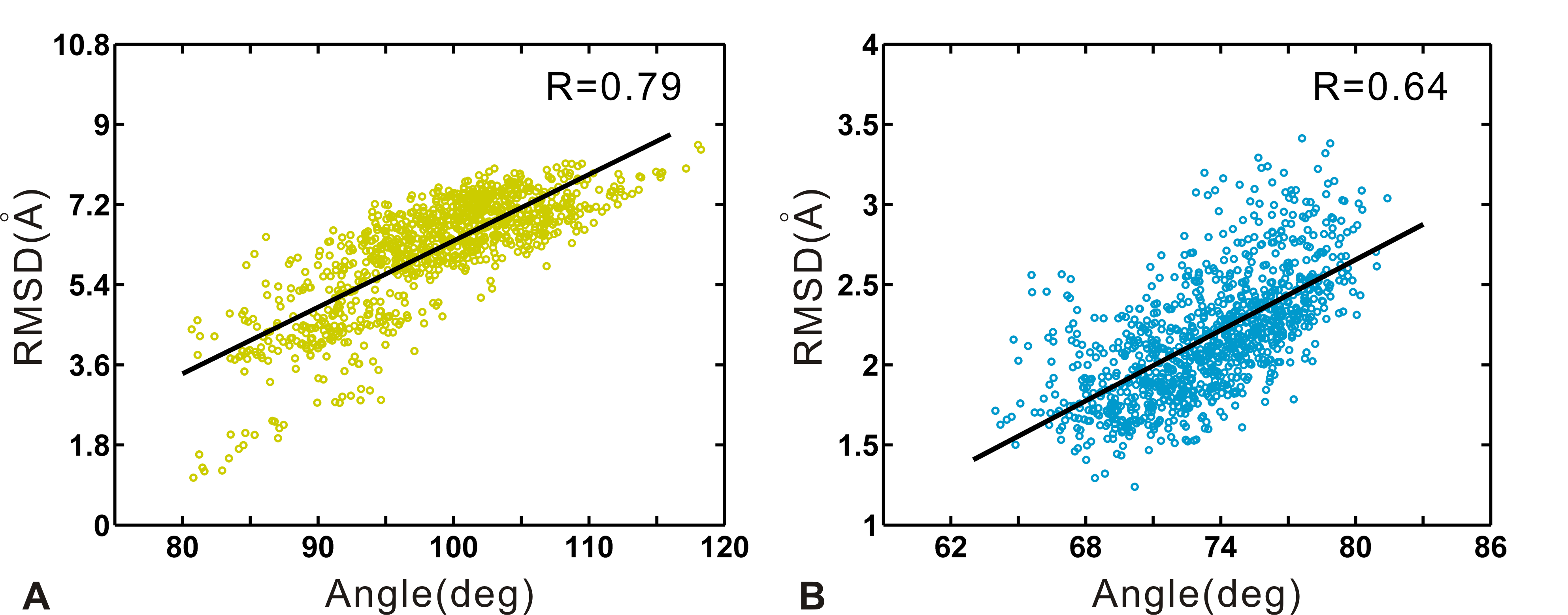

Supplement: Figure S2 — Correlations between the values of intramolecular angle and the RMSDs of the backbone atoms for the DNA-free (A) and DNA-bound (B) dHax3. The correlation coefficients are 0.79 for the DNA-free dHax3 and 0.64 for the DNA-bound dHax3, respectively. (TIF) [file pone.0076045.s002.tif]

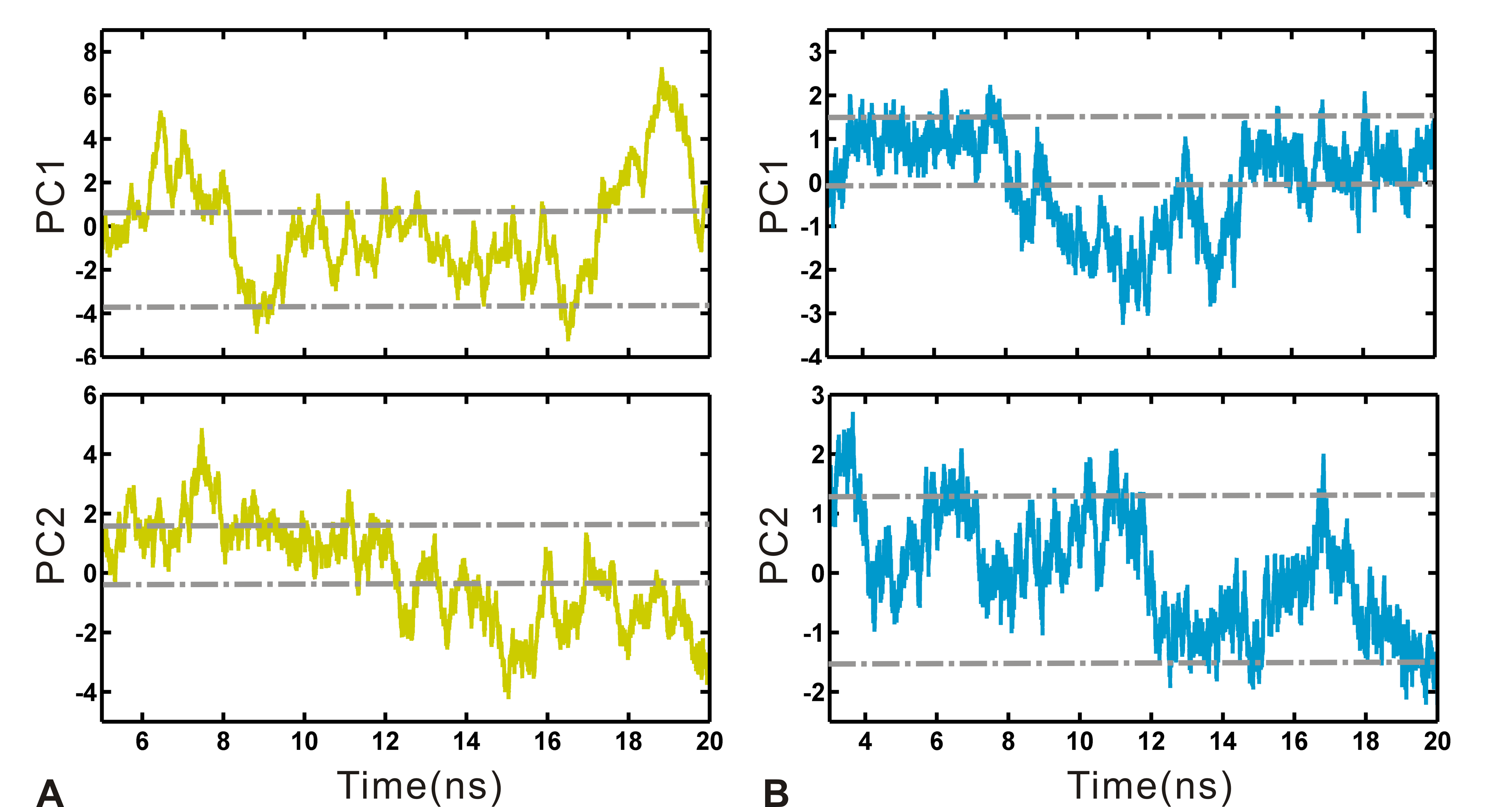

Supplement: Figure S3 — The principal components PC1 and PC2 versus simulation time in the DNA-free (A) and DNA-bound (B) systems. For the free-energy minima in the DNA-free system, the values of PC1 vary from −3.8 to 0.8 and those of PC2 from −0.3 to 1.6 (see Figure 7 A). For the free-energy minima in the DNA-bound system, the values of PC1 vary from 0 to 1.5 and those of PC2 from −1.5 to 1.3 (see Figure 7 B). Then, the time sections can be determined by searching the corresponding intervals of PC1 and PC2 (dotted line). Approximately, the free-energy minima in the DNA-free system (yellow) correspond to the segments of 9∼13 ns and 16∼18 ns, while those in the DNA-bound system (sky blue) correspond to the segments of 4∼8 ns and 15∼20 ns. (TIF) [file pone.0076045.s003.tif]
